# Recurrent atypical ductal hyperplasia in the setting of gynecomastia in a male patient: a unique finding and brief literature review

**DOI:** 10.1093/jscr/rjag713

**Published:** 2026-08-14

**Authors:** Amanda Rose Camarda, Vincent Marcucci, William P Frampton, Roshani Patel

**Affiliations:** St. George’s University School of Medicine, True Blue, St. George, Grenada; Department of Surgery, Jersey Shore University Medical Center, Neptune, NJ 07756, United States; Department of Pathology, Jersey Shore University Medical Center, Neptune, NJ 07756, United States; Department of Surgery, Jersey Shore University Medical Center, Neptune, NJ 07756, United States

**Keywords:** atypical ductal hyperplasia, ductal carcinoma *in situ*, true gynecomastia, nipple-sparing mastectomy, nipple–areola complex

## Abstract

Gynecomastia (GM) is a benign condition involving glandular proliferation within male breast tissue. Atypical ductal hyperplasia (ADH) is considered a high-risk lesion due to its association with an increased risk of breast cancer; however, the literature remains sparse regarding the male population with underlying ADH. ADH in the setting of GM. Currently, the reported incidence of ADH with GM ranges from 0.4% to 4.5%. Here, we present a case of a male patient with recurrent ADH after a previous excision for bilateral ADH that was upstaged to ductal carcinoma *in situ* (DCIS) after definitive surgery for the recurrent ADH. The ADH and DCIS were successfully excised via central lumpectomy. A brief literature review on its incidence and treatment within the male population was also conducted. This case highlights the rarity of ADH and DCIS in men, as well as their recurrence following nipple-sparing mastectomy.

## Introduction

Gynecomastia (GM) is a benign condition involving glandular proliferation within male breast tissue [1]. While it is common in infancy, adolescence, and in middle-aged to older males, this case report focuses on its presentation in the middle-aged male population. Rather than glandular proliferation, pseudogynecomastia (PG) occurs secondary to excessive fat deposition within male breast tissue and is often seen in those with obesity [1]. Atypical ductal hyperplasia (ADH) is considered a high-risk lesion due to its association with an increased risk of breast cancer; however, the literature remains sparse regarding the male population with underlying ADH in the setting of GM. Currently, the reported incidence of ADH in specimens containing GM ranges from 0.4% to 4.5% [2, 3]. In women, ~10%–25% of lesions histologically interpreted as ADH are upgraded to ductal carcinoma *in situ* (DCIS) or invasive cancer at the time of excision; therefore, surgical excision is routinely recommended for lesions showing ADH on core biopsy [4, 5]. Herein, we present a case of a male patient with recurrent ADH after resection for GM that was upstaged to DCIS, as well as a brief literature review on its incidence and treatment within the male population.

## Case presentation

The patient is a 41-year-old male patient who, in 2018, 6 years prior to his presentation to our clinic, had excision for left breast GM. He was found to have extensive ADH. Tomosynthesis did not demonstrate flame-like tissue within the left breast consistent with GM. He underwent re-excision/completion subareolar mastectomy of the left breast tissue and there was no ADH. He also had removal of prominent subareolar right breast tissue and there was ADH. The margins were negative for ADH. He underwent genetic testing in 2018 and the testing was negative. He has a family history of breast cancer in his maternal great-great-grandmother. There is no family history of other cancers. Sonography of the left breast for focal pain in 2020 and 2021 was unremarkable. He was not placed on tamoxifen, as it was felt to be of little benefit. In the Spring of 2024, the patient presented to our office with complaints of xeroderma to the left nipple without mass effect or drainage. Prior to presentation, the patient tried topical clobetasol and triamcinolone under the direction of an outside dermatologist with minimal symptomatic improvement.

Physical examination showed a raised, hyperpigmented area measuring 5 mm of xeroderma of the left breast at the 6 o’clock position to the areolar border, which is shown in Fig. 1. He was maintained on triamcinolone for a total of 2 weeks with no resolution. A punch biopsy of the left areola was performed, which demonstrated ADH.

**Figure 1:**
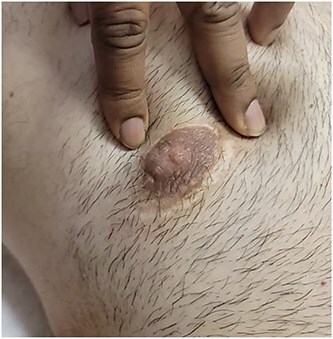
Gross image of the left nipple with small area of raised, hyperpigmented dry skin to the distal aspect of the nipple-areola border.

After further discussion, the decision was made to proceed with a left central lumpectomy under ultrasound guidance. The specimen was sent for permanent pathology, and the left breast tissue was ultimately upstaged to DCIS (Fig. 2). Final pathology reported DCIS, cribriform type, nuclear Grade 1, of the left breast spanning at least 2.0 mm on one slide (Fig. 3); there was no ADH. The patient’s postoperative course was uneventful, and his surgical incisions healed well.

**Figure 2:**
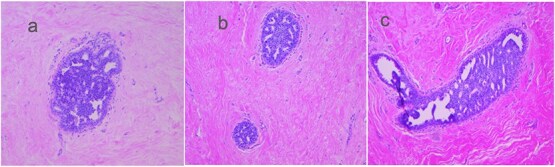
(a) Hematoxylin and eosin stain showing atypical ductal proliferation exhibiting a cribriform growth pattern; (b) hematoxylin and eosin stain showing two foci of atypical cribriform intraductal proliferations (resection specimen with DCIS); and (c) hematoxylin and eosin stain showing cribriform DCIS, nuclear Grade 1.

**Figure 3:**
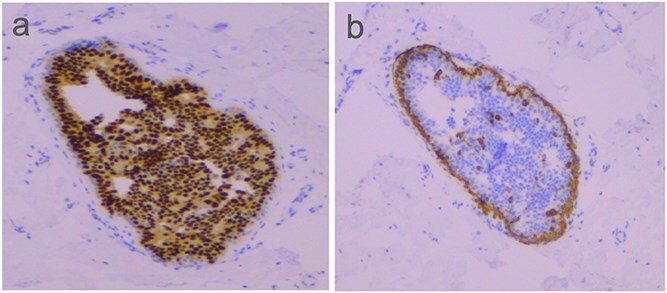
(a) Estrogen receptor stain showing uniform staining within an intraductal proliferation; (b) cytokeratin 5/6 stain showing absence of staining in lesional cells; this staining pattern does not help distinguish ADH from DCIS but will differentiate both lesions from usual duct hyperplasia.

## Discussion

True gynecomastia (TG) and PG are both benign pathologies involving male breast tissue; however, TG is secondary to glandular proliferation while PG is not [1]. The epidemiologic distribution of GM in the male population can be trimodal with peaks at neonatal infancy (0–30 days), early adolescence (12–14 years), and late adulthood-elderly (50–80 years) [6]. GM seen in both the neonatal and pubertal periods is often physiologic in nature, whereas GM seen in older adults and the elderly is more often pathological [1]. Neonatal GM is transient and often resolves after the first few weeks of life [7]. It is believed to be secondary to dehydroepiandrosterone (DHEA) and DHEA sulfate conversion to estrone and estradiol by the placenta. These estrogens enter the fetal circulation resulting in glandular proliferation of the breast tissue [7]. Pubertal GM is largely considered temporary, secondary to the temporary imbalance of estrogen and androgen that occurs during puberty [8]. More common post-pubertal causes of GM in younger adult males include alcohol and recreational drug use, including marijuana (≥4 times per week) and anabolic steroids [1, 8]. Other causes include chronic liver or kidney disease, malnutrition, hyperthyroidism, hypogonadism, testicular tumors, and human chorionic gonadotropin (hCG)-producing tumors [1]. Medications known to be associated with GM are summarized in Table 1.

**Table 1:** Drugs associated with gynecomastia.^a^

Mechanism	Drug name	Drug class
Estrogen-containing or estrogen-acting^b^	Digitalis	Cardiac glycoside
	Anabolic steroids	Drug of abuse
Endogenous estrogen enhancers^b^	Gonadotropins	Hormonal drug
	Clomiphene	Hormonal drug
Testosterone inhibitors^b^	Ketoconazole	Azole antibiotic
	Metronidazole	Nitroimadazole antibiotic
	Cimetidine	Histamine H2 antagonist
	Alcohol	Drug of abuse
	Marijuana	Drug of abuse
Hyperprolactinemia	Isoniazid	Antituberculosis drug
	Verapamil	CCB^c^
	D-amphetamine	Amphetamine
	Diazepam	Benzodiazepine
	Haloperidol	FGA^d^
	Risperidone	SGA^e^
	Omeprazole	PPI^f^
	Clomipramine	TCA^g^
	Amitriptyline	TCA

^a^Braunstein.

^b^Can lead to estrogen:testosterone imbalance.

^c^Calcium-channel blockers.

^d^First-generation antipsychotic.

^e^Second-generation antipsychotic.

^f^Proton-pump inhibitor.

^g^Tricyclic antidepressants.

ADH is a high-risk pathology characterized by intraductal clonal epithelial cell proliferation that can be associated with GM [9]. In females, ADH is identified in 3%–5% of breast biopsies, most often between 40 and 50 years of age. ADH in males is much less common and is identified in <1% of those with GM [10].

A brief literature review was conducted to analyze the management recommendations of ADH and DCIS in the male population, as they differ from those in the female population. The results of our review are summarized in Table 2. In some plastic surgery literature, pathologic examination of tissue is not always recommended. However, in one study, pathologic examination was suggested for high-risk patients. High-risk patients are categorized as those with ≥1 of the following factors: age, risk factors (identified as obesity, estrogen/steroid therapy, family history of breast cancer, and marijuana use), unilateral/bilateral location, estrogen/progesterone receptor positivity, tumor type, breast cancer gene 1 and 2 (BRCA1/BRCA2) positivity, and chromosomal analysis [11]. The current standard of care as followed by our institution is to send all specimens to pathology for examination.

**Table 2:** Summary of management recommendations during literature review.

Literature author(s)	Topic	Recommendations/conclusions
Coopey *et al*. [2]	ADH and its progression to breast cancer	No progression of ADH to breast cancer - ADH poses less of a risk in males compared to females - Surgical excision of GM-ADH tissue is curative of any potentially associated breast cancer risk
Agostini *et al*. [11]	Tumor incidence following pathologic examination according to six factors to justify pathologic analysis of GM specimens: - Age - Risk factors - Unilateral/bilateral location - Estrogen/progesterone receptor positivity - Tumor type - BRCA1/BRCA2 positivity and chromosomal analysis	Specimens should be sent for pathologic examination in **high-risk patients with ≥1 of the six factors**
Shirah and Shirah [15]	DCIS in GM	While GM is a benign pathology of the male breast, DCIS is a malignant variant, therefore **histopathological examination of resected GM should be carried out in all patients, regardless of age group**

While ADH has been known to increase the risk of breast cancer in women, little is known about breast cancer risk associated with ADH in men. A study focusing on a 6-year follow-up of ADH found in men (*n* = 14) who had surgical resection for GM, there was no reported progression to breast cancer, thus suggesting that ADH in males poses less of a risk than in females [2]. Interestingly, 10/14 patients in this study were noted to have bilateral ADH. Management remains similar in the male and female populations, suggesting surgical excision of ADH for curative and preventive measures [2].

Our patient underwent prior surgical resection with a nipple-sparing procedure (i.e. subcutaneous mastectomy) for GM removal. With the advancement in the evolution of breast surgery in women with breast cancer, compared to total mastectomy, nipple-sparing mastectomy has significant advantages including enhanced cosmesis, improved body image, and overall patient satisfaction. In a study of 126 female patients with nipple- or areolar-sparing mastectomy, ADH at the nipple margin emerged as an independent factor [12].

Breast cancer in men is rare, accounting for approximately <1% of the annual cancer burden worldwide compared to 12% in females [13]. DCIS of the breast is a lesion confined within the mammary ducts, without invasive features. Pure DCIS comprises ~10% of breast cancer in the male population and <0.1% of overall cancers observed in men [14]. In a retrospective analysis of 74 male patients with GM (five of whom were found to have DCIS), it was noted that while GM is a benign pathology of the male breast, DCIS is a malignancy; therefore histopathological examination of resected GM should be carried out in all patients, regardless of age group [11, 15].

In conclusion, our report, with a case presentation in conjunction with review of existing literature, highlights the rarity of ADH occurrence in males and how its presentation and management differ compared to the female population. Ultimately, we conclude that ADH in males is best managed by histopathological examination with resection to reduce the burden of future occurrence of malignancy and provide optimal outcomes for male patients. However, more research is needed to further investigate the differences in this pathology among males and females.
